# Case Report: Image-guided adaptive lattice radiotherapy for a 2,772-cc ulcerative triple-negative breast cancer

**DOI:** 10.3389/fonc.2026.1942852

**Published:** 2026-09-22

**Authors:** Min Xiao, Qian Wang, Gangyang Yu, Fang Li, Jingna Ji

**Affiliations:** Department of Radiation Oncology, Hankou Hospital Affiliated with Wuhan University of Science and Technology (Wuhan Hankou Hospital), Wuhan, Hubei, China

**Keywords:** adaptive radiotherapy, giant ulcerative breast cancer, lattice radiotherapy, spatially fractionated radiotherapy, triple-negative breast cancer

## Abstract

**Background:**

Giant ulcerative breast cancers (>2,000 cc) with active hemorrhage present a critical therapeutic challenge, as conventional radiotherapy carries a high risk of severe toxicity to surrounding organs. Lattice radiotherapy (LRT) delivers spatially fractionated high-dose vertices within a low-dose background, offering the potential to overcome dose constraints. However, rapid tumor regression induced by LRT may render fixed plans unsafe, necessitating adaptive replanning.

**Case presentation:**

A 46-year-old female with stage IV triple-negative breast cancer (TNBC; BRCA1 germline mutation) presented with a 2,772.2-cc ulcerative left breast and axillary masses complicated by persistent hemorrhage, recurrent infection, and Eastern Cooperative Oncology Group (ECOG 3). After failure of systemic therapies including fluzoparib, image-guided adaptive LRT was initiated (gross tumor volume [GTV]: 12 Gy/4 fractions; lattice vertices: 60 Gy/4 fractions). Before fractions 2–4, the GTV was re-contoured on daily fan-beam computed tomography (FBCT), lattice vertices were redistributed away from the skin surface, and plans were recalculated. LRT was followed by conventionally fractionated VMAT (40 Gy/20 fractions). Hemorrhage ceased within 2 weeks and persistent fever subsided, becoming intermittent and progressively less frequent. Tumor volume progressively decreased by 21.2% during LRT (before fraction 4), 81.5% post-VMAT, and 96.1% (108.3 cc residual) at 2-month follow-up. Cumulative organ-at-risk (OAR) doses, estimated by rigid-registration dose accumulation, remained within tolerance, with only Grade 2 dermatitis and no ≥Grade 3 toxicity.

**Conclusion:**

This case demonstrates the technical feasibility of image-guided adaptive lattice replanning in a rapidly changing, ultra-large fungating breast tumor. Adaptive replanning may reduce geometric displacement of high-dose vertices; however, its dosimetric and clinical benefits require confirmation in further patients and with longer follow-up.

## 1 Introduction

Advanced breast cancer with giant fungating masses (>2,000 cc) complicated by ulceration, hemorrhagic exudate, and infection poses an extraordinarily difficult clinical scenario (1, 2). Such patients typically present with ECOG PS 3–4 and are often unfit for radical surgery or intensive chemotherapy due to severe anemia, high sepsis risk, and poor general condition (3). Although conventional radiotherapy can achieve hemostasis, delivering an effective biological dose across such an enormous target volume inevitably exceeds the tolerance of the ipsilateral lung, heart, brachial plexus, and chest wall skin, risking radiation pneumonitis or chest wall necrosis (4–6). Therefore, the primary goal is not immediate tumor ablation but urgent control of hemorrhage and infection—stabilizing an uncontrolled wound crisis to create a therapeutic window for subsequent interventions.

LRT creates a three-dimensional heterogeneous dose distribution composed of high-dose peaks (vertices) and low-dose valleys, thereby circumventing the dose constraints of conventional uniform irradiation; it is currently applied primarily to solid tumors >5 cm in diameter. The technique delivers ultra-high doses to the tumor core while simultaneously protecting surrounding OARs (7). Biologically, high-dose vertices induce immunogenic cell death and microvascular disruption, while low-dose valleys may exert bystander and potentially abscopal effects on distant metastases (8–10)—a property of particular interest in widely disseminated disease.

Our patient presented with a giant breast tumor and massive axillary lymphadenopathy, accompanied by persistent hemorrhagic exudate and recurrent wound infection. Conventional antitumor and supportive therapies failed to control these complications, prompting LRT. During treatment, rapid tumor collapse was observed. Had the original plan been maintained, the high-dose vertices would have deviated from the hemorrhagic core and approached the skin, creating a serious safety hazard. Consequently, an image-guided adaptive LRT workflow was implemented. This report presents this critical case (~2,772 cc) in detail, demonstrating daily CT-based target re-contouring and high-dose vertex redistribution, aiming to provide a reference dosimetric paradigm for palliative radiotherapy of similar uncontrolled fungating wounds. The treatment intent was urgent palliative hemostasis, with adaptive LRT serving as a bridge to consolidation radiotherapy and ongoing systemic therapy.

## 2 Case presentation

### 2.1 Patient history and baseline characteristics

A 46-year-old woman first noticed a left breast mass approximately 9 months before presentation but did not seek medical attention. The mass rapidly enlarged to approximately 14 cm in greatest dimension. In January 2026, she presented to our institution. Chest CT revealed a left breast mass suspicious for malignancy, with bilateral axillary, supraclavicular, and internal mammary lymph node metastases; multiple bone metastases; bilateral pleural effusion with atelectasis; bilateral pulmonary metastases; and a left adrenal metastasis. Contrast-enhanced MRI of the head, abdomen, and pelvis confirmed these findings and additionally revealed extensive retroperitoneal and abdominopelvic metastases.

Left breast mass core needle biopsy, genetic testing, and pleural fluid cytology established the diagnosis: invasive ductal carcinoma, triple-negative phenotype (ER-/PR-/HER2-), Ki67 labeling index 80%, histological grade 3, with a BRCA1 germline pathogenic mutation. Family history: both parents alive and well, no siblings, and no family history of cancer or hereditary disease; the BRCA1 germline mutation was therefore presumably *de novo*. The patient was married and cared for by her husband and son; the exophytic, malodorous, intermittently hemorrhaging wound had confined her largely to bed and caused marked social withdrawal and psychological distress. On admission for radiotherapy (March 16, 2026), physical examination revealed: temperature 37.8 °C, pulse 90/min, respiration 22/min, and blood pressure 140/75 mmHg; the patient was conscious and cooperative but showed an anemic appearance and was transported by trolley; on specialist examination, a giant, hard, ill-defined left breast mass (approximately 14 × 12 × 6 cm) with erosion, ulceration, hemorrhage, and foul odor involved the nipple-adjacent skin and was tender on pressure; a giant left axillary mass with similar ulceration and hemorrhage was present, with left arm edema preventing elevation; enlarged bilateral supraclavicular lymph nodes were palpable. (Clinical photographs are provided in **Figure 1**).

**Figure 1:**
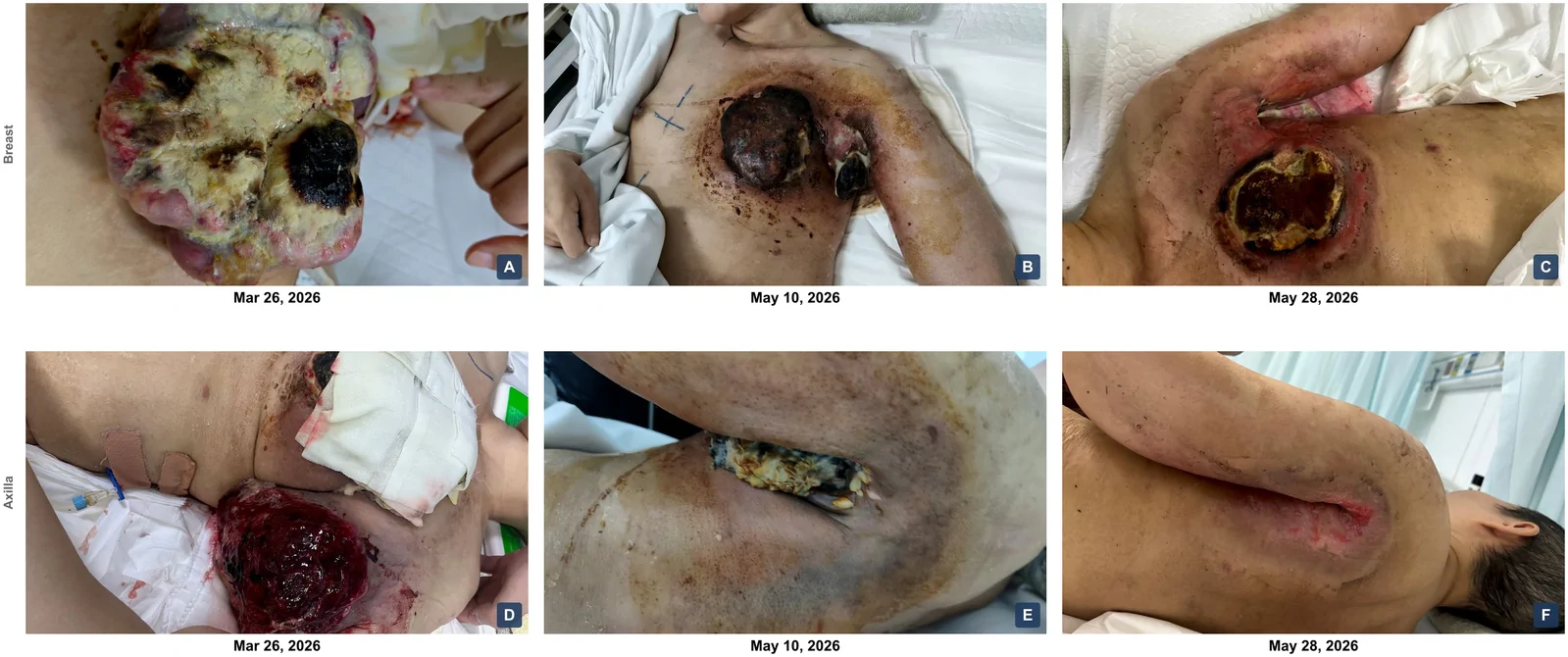
Clinical photographs demonstrating the response of the ulcerative tumor masses to adaptive lattice radiotherapy and conventionally fractionated VMAT. Upper row (breast) **(A)** Baseline (March 26) showing extensive ulceration and hemorrhagic crusting; **(B)** end of VMAT (May 10) with marked wound drying and shrinkage; **(C)** 18 days post-treatment (May 28) showing continued healing. Lower row (axilla) **(D)** Baseline (March 26) with active hemorrhagic exudate and foul odor; **(E)** end of VMAT (May 10) with cessation of bleeding and exudate; **(F)** 18 days post-treatment (May 28) with near-complete wound healing.

Following diagnosis, denosumab 120 mg every 4 weeks was initiated on February 4, 2026, for bone metastases. One cycle of albumin-bound paclitaxel 100 mg/m² was administered on February 6, 2026; however, subsequent chemotherapy was withheld due to fever and anemia. Left intrapleural bevacizumab 200 mg perfusion was performed twice (February 11 and March 5, 2026). Oral fluzoparib (a PARP inhibitor) was initiated on February 13, 2026 at 50 mg twice daily, escalated to 100 mg twice daily on February 21 and 150 mg twice daily on March 1; it was continued throughout radiotherapy, held on April 26 for leukopenia, and resumed at discharge. Left intrapleural carboplatin 200 mg perfusion was administered on March 27, 2026, between LRT fractions 1 and 2. Throughout the disease course, repeated red blood cell transfusions and broad-spectrum antibiotics were given for severe anemia and recurrent wound infection.

Despite these interventions, the patient continued to experience persistent fever (maximum 38.7 °C), with erosion, persistent hemorrhage, and foul odor from both the left breast and axillary masses, as well as marked left upper extremity lymphedema with limited mobility. At the initiation of radiotherapy planning, hemoglobin was 67 g/L (March 23, 2026) despite repeated transfusion. Her ECOG was 3, with a Numerical Rating Scale (NRS) pain score of 1. Given the anticipated inadequacy of conventional radiotherapy for such a massive tumor, and in accordance with the patient’s primary treatment goal of rapid hemorrhage control, LRT was adopted to prioritize control of the hemorrhage and recurrent infection.

### 2.2 LRT treatment planning and implementation

Simulation and target delineation: The patient underwent simulation on a uLinac ETernaTx linear accelerator (United Imaging, China) in the supine position, immobilized with a vacuum cushion. A 1-cm tissue bolus was applied over the left breast and axillary mass to enhance the surface dose. The bolus was a 30 × 30-cm thermoplastic elastomer sheet that conformed well to the exophytic tumor surface; the wound was covered with a single layer of sterile gauze, and alignment marks drawn on the surrounding skin allowed daily reproduction of bolus placement. Excess air was pressed out manually at each application; residual bolus–skin air pockets over the large exophytic mass were unavoidable and were therefore manually contoured on each planning/FBCT image and assigned air density for dose correction. Planning CT images were acquired at 3-mm slice thickness and transferred to the uTPS treatment planning system (United Imaging, China). Plans were calculated in the uTPS with a Monte-Carlo dose-calculation algorithm on a 0.2-cm grid and delivered with four tangential segmented VMAT arcs at 6 MV in flattening-filter-free mode. The GTV—comprising the combined left breast mass and left axillary mass—measured 2,772.2 cc. A structure designated GTVsub1cm was generated by isotropic internal contraction of the GTV by 1 cm. OARs included bilateral lungs, heart, left brachial plexus, spinal cord, skin defined as a manually contoured 5-mm rind of the external surface (including the tumor-infiltrated and ulcerated regions), and contralateral breast.

Lattice vertex configuration and dose prescription: Lattice vertices (lattice target volume, LTV) of 1.5-cm diameter were arranged in a three-dimensional grid within the GTVsub1cm, with center-to-center spacing of 3–4 cm, avoiding blood vessels and the brachial plexus. The dose prescription was: GTV 12 Gy in 4 fractions; LTV 60 Gy in 4 fractions, D95-LTV (dose received by at least 95% of the volume) ≥ 1,500 cGy. The ablative dose ratio (ADR, LTV volume/GTV volume) was maintained at 1%–10%, and the lattice density index (LDI, number of vertices per 100 cc of GTV volume) at 1–8. Treatment was delivered using volumetric modulated arc therapy (VMAT) with four tangential segmented arcs at 6 MV in flattening-filter-free (FFF) mode at 1,400 MU/min on the uLinac ETernaTx, with daily FBCT image guidance. **Supplementary Figure S1** shows the dose distributions of the LRT and subsequent conventionally fractionated VMAT plans.

### 2.3 Image-guided adaptive LRT workflow

FBCT before fraction 2 revealed rapid tumor regression. As the tumor collapsed, several fixed vertices from the initial plan deviated from the hemorrhagic core and approached the skin, risking skin overdose and loss of hemostatic efficacy. **Figure 2** illustrates the vertex positions before and after redistribution, along with a paired dosimetric comparison.

**Figure 2:**
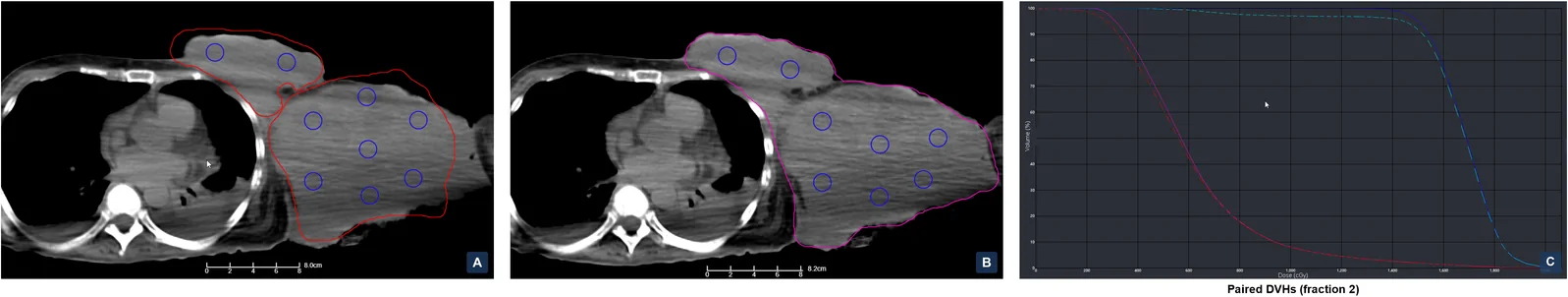
Image-guided adaptive lattice replanning and paired dosimetric comparison. **(A)**​ FBCT acquired before the second fraction, showing four lattice vertices superior to the tumor located close to the body surface. The blue circular contour indicates the pre-adjustment lattice target volume (LTV), and the red contour indicates the pre-adjustment gross tumor volume (GTV). **(B)**​ FBCT after redistribution of the lattice vertices, demonstrating repositioning away from the skin and concentration within the residual tumor core. The blue circular contour indicates the post-adjustment LTV, and the magenta contour indicates the post-adjustment GTV. **(C)**​ Dose–volume histograms before and after replanning for fraction 2: solid lines denote the adapted LTV (deep blue) and GTV (rose); dashed lines denote the non-adapted LTV (light blue) and GTV (red).

Given the patient’s poor condition—each setup required staff-assisted transfer, and prolonged immobilization was not tolerated—replanning was performed off-couch (patient returned to the ward during each session). The workflow comprised (1): FBCT acquisition with rigid registration to the planning CT (2); GTV re-contouring on the current FBCT (3); deletion or repositioning of vertices near the skin (4); plan recalculation and measurement-based patient-specific QA (ArcCheck, 3%/3 mm, action level ≥95%; **Table 1**) (5); re-setup, verification FBCT for image-guided correction, confirmation of body surface, bolus, dressings, and limb position, followed by delivery. This cycle was repeated for fractions 2–4 (March 26, 31, April 3, and 7, 2026). For fraction 2, the FBCT was acquired on the afternoon of March 30. The physician then adjusted the target contours and lattice spheres, and the physicist modified the plan accordingly. The entire replanning process, including patient-specific QA, took approximately 4 hours. The adapted plan was delivered on the morning of March 31, following pre-treatment verification FBCT. Fractions 3–4 used morning FBCT with ~3 h replanning and same-afternoon delivery. Dosimetric parameters are in **Table 1**. To preserve the peak–valley distribution, the previous fraction’s spheres were copied onto the current FBCT after rigid registration; only peripheral vertices near the shrinking target edge or skin were deleted or translated, maintaining the intended cumulative 60-Gy peaks. The workflow with timestamps is in **Supplementary Figure S2**.

**Table 1:** Dosimetric comparison between non-adapted and adapted lattice plans for fractions 2–4.

Parameter	Fraction 1	Fraction 2		Fraction 3		Fraction 4	
		Non-adapted	Adapted	Non-adapted	Adapted	Non-adapted	Adapted
N-lattice (vertices)	32	32	26	26	25	25	23
V-GTV (cc)	2772.2	2772.2	2531.7	2531.7	2442.5	2442.5	2185.1
V-LTV (cc)	64.86	64.86	49.32	49.32	46.25	46.25	45.74
LDI (per 100 cc)	1.15	1.15	1.03	1.03	1.02	1.02	1.05
ADR (%)	2.34	2.34	1.95	1.95	1.89	1.89	2.09
Dmean-GTV (cGy)	766.87	608.43	623.52	638.10	645.23	687.3	713.71
D95-GTV (cGy)	414.80	277.85	316.48	348.92	327.70	355.84	313.22
Dmean-LTV (cGy)	1686.33	1626.38	1687.72	1659.25	1667.49	1624.33	1655.67
D98-LTV (cGy)	1450.56	754.68	1451.82	1428.03	1442.21	1372.80	1446.07
D95-LTV (cGy)	1500	1435.48	1500	1471.50	1500	1427.23	1500
D50-LTV (cGy)	1672.25	1679.05	1691.50	1651.26	1660.13	1630.08	1662.23
D2-LTV (cGy)	1880.43	1917.31	1918.49	1820.09	1863.50	1834.22	1887.90
D1cc-Skin (cGy)	853.10	1442.95	661.74	1002.35	692.33	1224.55	615.42
D2cc-Skin (cGy)	761.03	1348.93	607.68	926.67	627.79	1137.98	579.44
Dmean-LTV/Dmean-GTV ratio	2.20	—	2.71	—	2.58	—	2.32
Total MU	11103	—	9376	—	8755	—	8124
Gamma pass rate (%)	99.9	—	99.8	—	99.9	—	99.6
Beam-on time (min)	8.6	—	7.8	—	7.2	—	6.7

LRT, lattice radiotherapy; N-lattice, number of lattice vertices; V-GTV, volume of gross tumor volume; V-LTV, volume of lattice target volume (sum of all vertex volumes); Dmean-LTV/Dmean-GTV ratio, mean LTV dose divided by mean GTV dose; LDI, lattice density index (number of vertices per 100 cc of GTV); ADR, ablative dose ratio (V-LTV/V-GTV); Dmean/D98/D95/D50/D2, dose received by at least the stated percentage of the structure volume; D1cc/D2cc-Skin, minimum dose to the hottest 1/2 cc of the skin structure; Non-adapted, the preceding fraction’s plan recalculated on the current-fraction FBCT anatomy without vertex redistribution; Adapted, the clinically delivered plan. Prescription criterion: D95-LTV ≥1,500 cGy (15 Gy per fraction).

Paired dosimetric comparison:​ for fractions 2–4, the preceding fraction’s plan (non-adapted) was recalculated on the current FBCT anatomy and compared with the delivered adapted plan. Target coverage and skin dose (D1cc/D2cc of a 5-mm external-surface rind) were evaluated. D0.03cc was not used, as a near-point maximum was considered unrepresentative for an irregular, ulcerated, dressing-covered surface. After completing 4 LRT fractions, a conventionally fractionated VMAT plan was implemented. The patient rested for 1 week and underwent a repeat simulation CT. A new GTV (GTV1) was contoured with a 0.5-cm margin to generate PGTV1, which was prescribed 40 Gy in 20 fractions (2 Gy/fraction), delivered from April 13 to May 10, 2026. Offline adaptive plan modifications were performed at the 7th and 16th fractions.

## 3 Results

### 3.1 Dosimetric benefit of adaptive replanning (paired comparison)

For fractions 2–4, the preceding non-adapted plan was recalculated on the current-fraction FBCT anatomy and compared with the delivered adapted plan (**Table 1**). Without adaptation, D95-LTV fell below the prescription criterion (≥1,500 cGy) in all three fractions (mean 1,444.7 cGy; 95.1–98.1% of prescription); adaptive vertex redistribution restored D95-LTV to 1,500 cGy in every fraction, with mean LTV D98 improving from 1,185.2 to 1,446.7 cGy. Skin D1cc would have reached 1,442.95, 1,002.35, and 1,224.55 cGy for fractions 2–4, respectively—approaching the full 15-Gy single-fraction vertex dose to 1 cc of skin—whereas the adapted plans limited these values to 661.74, 692.33, and 615.42 cGy (reductions of 54.1%, 30.9%, and 49.7%; mean 44.9%). Corresponding D2cc reductions ranged from 32.3% to 55.0%. The minimum vertex–skin distance was 2 mm. All plans passed patient-specific QA (gamma 3%/3 mm; 99.6–99.9%), and beam-on time decreased from 8.6 min (fraction 1) to 6.7 min (fraction 4).

### 3.2 Organ-at-risk doses and cumulative estimation

OAR doses are reported separately for each treatment phase because they were computed on different simulation anatomies. For the 4-fraction LRT plan summation (image registration onto the planning CT): left lung V20 = 0%, mean dose 511.04 cGy; heart mean dose 290.69 cGy; chest wall V30 = 0.14%; brachial plexus D0.03cc = 759.25 cGy. For the 20-fraction VMAT dose evaluation (re-simulation CT anatomy; including two adaptive modifications): left lung V20 = 10.77%, mean dose 670.25 cGy; heart mean dose 139.25 cGy; chest wall V30 = 8.25%; brachial plexus D0.03cc = 4029.71 cGy.

For cumulative estimation, all seven plans (four LRT and three VMAT plans) were registered by bone-based rigid registration to the final VMAT plan CT and dose-summated on this common reference anatomy: heart mean dose 372.08 cGy; left lung mean dose 705.46 cGy, V20 = 3.82%. The cumulative V20 is lower than the VMAT-phase value because the reference anatomy (post-regression, with lung re-expansion) has a larger lung volume, and rigid registration partially smears the high-dose region. As a conservative upper bound, direct numerical summation yields a left lung mean dose of ≤11.8 Gy and V20 ≤10.8%; the true cumulative dose lies between these estimates and was reviewed slice-by-slice, with OAR evaluation following AAPM Task Group 101 Report (11) and the UK consensus on normal-tissue dose constraints for stereotactic ablative body radiotherapy (12). Brachial plexus: on retrospective review by two senior radiation oncologists, the plexus was not encased within the GTV, although a portion lay within PGTV1 after the 0.5-cm expansion. The estimated cumulative dose (on the common reference anatomy) was 4671.2 cGy, corresponding to an EQD2 of approximately 47.7 Gy (α/β = 3 Gy), below the 60–66 Gy tolerance range. Overall, both phase-specific and cumulative OAR estimates indicate that all organs at risk, including the left lung, heart, chest wall, and spinal cord, remained within tolerance limits.

### 3.3 Treatment response

The therapeutic response was consistent with the multimodal strategy of adaptive LRT, conventionally fractionated VMAT, and concurrent systemic therapy. Following the initial 4-fraction adaptive LRT (March 26–April 7, 2026), tumor volume decreased from 2,772.2 cc to 2,185.1 cc (FBCT before fraction 4; 21.2% reduction), with cessation of hemorrhagic exudate. During the subsequent VMAT course, regression continued: volumes were 1,493.9 cc on April 13 (46.1% reduction), 1,063.1 cc on April 21 (61.7%), and 668.1 cc on May 5 (75.9%). At VMAT completion (May 10), volume was 512.7 cc (81.5%); the decrease during the 1-week interval between LRT and VMAT (2,185.1 to 1,493.9 cc) likely reflects the delayed effect of LRT. Two months after both radiotherapy courses, follow-up chest CT showed further reduction to 108.3 cc (96.1% from baseline), with minimal residual radiographic mass—no metabolic or pathological confirmation was obtained, and this volume may include necrosis and post-treatment changes. Tumor volumes were measured on FBCT/CT (3-mm slices) by semi-automatic segmentation with manual editing. **Figure 3** illustrates the serial CT images and the volume regression curve.

**Figure 3:**
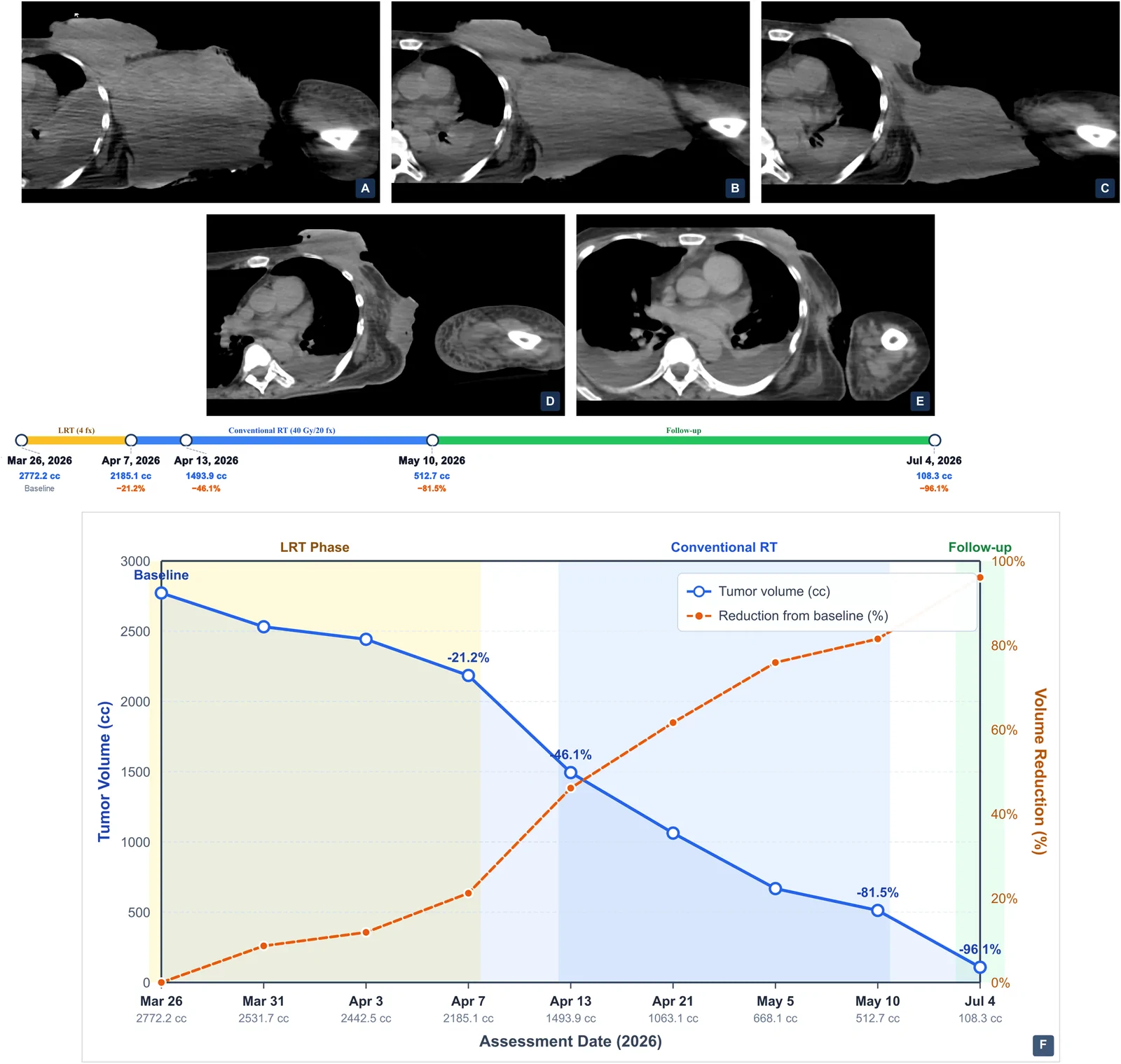
Serial computed tomography (CT) images and tumor volume regression during treatment. **(A–E)** Axial CT at key time points: baseline (March 26, 2026; 2,772.2 cc); after 4-fraction adaptive LRT (April 7; 2,185.1 cc); at the start of conventionally fractionated VMAT (April 13; 1,493.9 cc); at the end of VMAT (May 10; 512.7 cc); and at two-month follow-up (July 4; 108.3 cc). All images are at comparable anatomical levels through the largest tumor cross-section. The colored timeline bar indicates the three phases: LRT (yellow), conventionally fractionated VMAT (blue), and follow-up (green). **(F)** Tumor volume (cc, left axis) and percentage regression from baseline (right axis) over time.

### 3.4 Toxicity and follow-up

Treatment was well tolerated with rapid clinical improvement. Adverse events were graded per CTCAE v5.0. Hemorrhage ceased completely within 2 weeks. Infection control: throughout the remainder of treatment, the patient no longer experienced persistent fever; fevers became intermittent and progressively less frequent, and antibiotic use decreased. Foul odor progressively diminished. Pain remained well controlled (NRS 1–4); after LRT, left upper extremity edema gradually resolved with improved range of motion. The patient developed Grade 2 acute radiation dermatitis involving the left chest wall, axilla, and medial upper arm, presenting as moist desquamation confined to skin folds and tumor-invaded areas. No Grade ≥3 toxicity occurred. Fluzoparib was held on April 26, 2026, for Grade 2 leukopenia and resumed at discharge. No clinical or imaging evidence of radiation pneumonitis, brachial plexopathy, or chest wall necrosis was noted during follow-up. However, a small amount of residual tumor tissue remained in the left breast, and a cavity formed in the left axilla after tumor regression; both wounds remained open and required regular dressing changes. As of the time of writing, the patient continues to receive maintenance treatment in our department, and both she and her family have expressed gratitude to the medical team for the symptom relief achieved. Written informed consent for publication, including clinical photographs, was obtained from the patient.

## 4 Discussion

Giant fungating ulcerative breast cancer is a distinct and challenging entity. Zagardo et al. highlighted heterogeneous treatment strategies, poor standardization, and dismal prognosis (3). Abdallah et al. reported a 42% wound closure complication rate (4). For our patient—stage IV TNBC with active hemorrhage and ECOG 3—radical surgery, systemic chemotherapy, or conventional radiotherapy alone were unsuitable. TNBC lacks ER, PR, and HER2, is insensitive to endocrine and anti-HER2 therapy, and carries a worse prognosis with limited treatment options (13, 14), making this case more challenging than hormone receptor-positive counterparts. Rapid hemostasis was the overriding priority, as persistent bleeding from a 2,700-cc wound can be life-threatening.

LRT offers unique advantages: high-dose vertices induce endothelial apoptosis, microvascular thrombosis, and stromal collapse, producing rapid hemostasis. Sang et al. reported modified LRT (15 Gy × 3, then 36 Gy/18 fractions VMAT) for a similar ulcerative breast cancer (≈2,600 cc), achieving hemostasis within 5 days (15). However, their patient was hormone receptor-positive/HER2-negative, with inherently better prognosis. Our TNBC patient with BRCA1 mutation achieved hemostasis within 2 weeks and 96.1% volume reduction at 2 months. These comparisons are descriptive; differences in subtype, systemic therapy, and follow-up preclude superiority conclusions. Nevertheless, this suggests that adaptive LRT combined with conventional radiotherapy may hold particular value in refractory breast cancer.

Reports on adaptive LRT for giant breast cancer are scarce. Sang et al. (15) adjusted plans after rapid regression but did not detail the workflow. In other sites, Dincer et al. used MR-Linac online adaptive LRT for liver metastases (16), limited by MR-Linac availability. Zhou et al. reported adaptive replanning for NSCLC after 3 LRT fractions, but adjustment occurred during subsequent radiotherapy, not during LRT (17). Knight et al. reported early clinical outcomes of forward-planned MLC-based SFRT for large, bulky tumors across multiple sites and suggested that this rapid technique could be combined with adaptive treatment, but lattice vertex redistribution was not detailed (18). Amendola et al. generated adaptive plans when CBCT showed volume reduction, but used single-fraction LRT with adjustments only to subsequent conventional radiotherapy (19).

Our case—breast mass with giant axillary mass, an exceptionally rare presentation—describes daily FBCT-guided target re-contouring, vertex redistribution, and plan recalculation. After one fraction, tumor collapse was evident. Without timely redistribution, vertices would have drifted toward the skin, compromising safety and hemostasis. Paired dosimetry confirmed this: adaptation reduced skin D1cc by 31–55% while restoring D95-LTV compliance. This supports the emerging consensus that adaptive replanning for large, rapidly responding tumors is a safety measure, not merely optimization.

Notably, at the latest follow-up, the axillary mass had nearly completely disappeared, whereas the residual 108 cc of tumor was predominantly located in the breast region. In addition to the higher lattice vertex density in the axillary region, the following factors may have contributed (1): the breast region was subject to dose constraints from the heart, lung, and skin, limiting lattice vertex placement and resulting in relatively insufficient effective dose (2); the axillary region has richer lymphatic drainage and higher immune infiltration density, potentially enhancing LRT-induced local immune effects (3); differences in blood supply, oxygenation, and interstitial pressure across anatomical sites may also influence radiosensitivity.

This study has several limitations. First, as a single case report, the generalizability of the findings is limited, and larger prospective studies are needed. Second, the latest follow-up was at 2 months post-treatment, a relatively short interval; data on the durability of local control, late radiation toxicity, and overall survival are not yet available. Third, due to the enormous tumor volume, on-line adaptive replanning—involving target re-contouring, vertex redistribution, and plan recalculation—was time-consuming, and online (same-session) replanning would have required prolonged immobilization that the patient could not tolerate; therefore, an offline adaptive mode was adopted, which did not achieve real-time online adjustment before each fraction. This compromise may have missed more subtle interfraction anatomical changes, and future exploration of automated planning tools to shorten the workflow is warranted. Fourth, FBCT-based dose calculation relied on the vendor-calibrated CT-to-electron-density curve; residual bolus–skin air pockets were manually contoured and density-corrected, but the ulcerated wound surface, dressings, and bolus may still compromise surface-dose accuracy. Fifth, cumulative OAR doses were estimated by bone-based rigid registration, which cannot fully account for deformation, tissue collapse, and cavity formation; deformable accumulation was considered unreliable and not attempted, and cumulative values should be interpreted within the stated bounds.

## 5 Conclusion

This study reports a 2,772-cc giant ulcerative triple-negative breast cancer with a BRCA1 mutation treated with image-guided adaptive lattice radiotherapy (LRT) followed by conventionally fractionated VMAT. At 2-month follow-up, the tumor volume was 96.1% smaller than baseline, and phase-specific and cumulative organ-at-risk estimates remained within tolerance, with no ≥Grade 3 toxicity. This case demonstrates the technical feasibility of image-guided adaptive lattice replanning in a rapidly changing, ultra-large fungating breast tumor. Adaptive replanning may help maintain appropriate high-dose vertex positioning during marked anatomical changes, although its dosimetric and clinical benefits require confirmation in further patients and with longer follow-up. To our knowledge, this is one of the first systematic descriptions of an offline adaptive LRT workflow based on daily FBCT-guided target re-contouring and lattice vertex redistribution in breast cancer. The paired dosimetric comparison confirmed substantially reduced skin dose and preserved vertex coverage versus the non-adapted plans, supporting the use of adaptive LRT as a bridging treatment for patients with ulcerated wounds that are difficult to control by other means.

## Data Availability

The raw data supporting the conclusions of this article will be made available by the authors, without undue reservation.

## Supplementary material

Supplementary Figure 1 Dose Distributions of the Lattice Radiotherapy (LRT) and Subsequent Conventionally Fractionated VMAT Plans.​ Upper row (LRT): The magenta curve represents the GTV, and the blue spheres represent the LTV. (A) Axial, (B) sagittal, and (C) coronal views showing the spatially fractionated dose distribution, with (D) dose cloud display. Lower row (VMAT plan): The blue curve represents the PGTV1. (E) Axial, (F) sagittal, and (G) coronal views, with (H) dose band display.

Supplementary Figure 2 Adaptive LRT replanning workflow. (A) The nine-step workflow performed before fractions 2–4: FBCT acquisition, rigid registration to the planning CT, GTV re-contouring, lattice vertex redistribution, plan recalculation (uTPS, 0.2-cm grid), ArcCheck patient-specific QA (gamma 3%/3 mm, action level ≥95%), re-setup with bolus/alignment-mark reproduction, verification FBCT with setup correction, and delivery (6 MV FFF, four tangential arcs).
